# Supplementary material for: Clinical Impact of the Geriatric Nutritional Risk Index on Chemotherapy-Related Adverse Events in Diffuse Large B-Cell Lymphoma: A Multicenter Study
Source: Nutrients. 2025 Dec 2;17(23):3785. doi: 10.3390/nu17233785 (PMC12694119; doi:10.3390/nu17233785)
Supplement: Supplementary file 1 [file nutrients-17-03785-s001.zip › nutrients-3960720-supplementary.pdf]

Supplementary figure 1. Flowchart for patient selection.

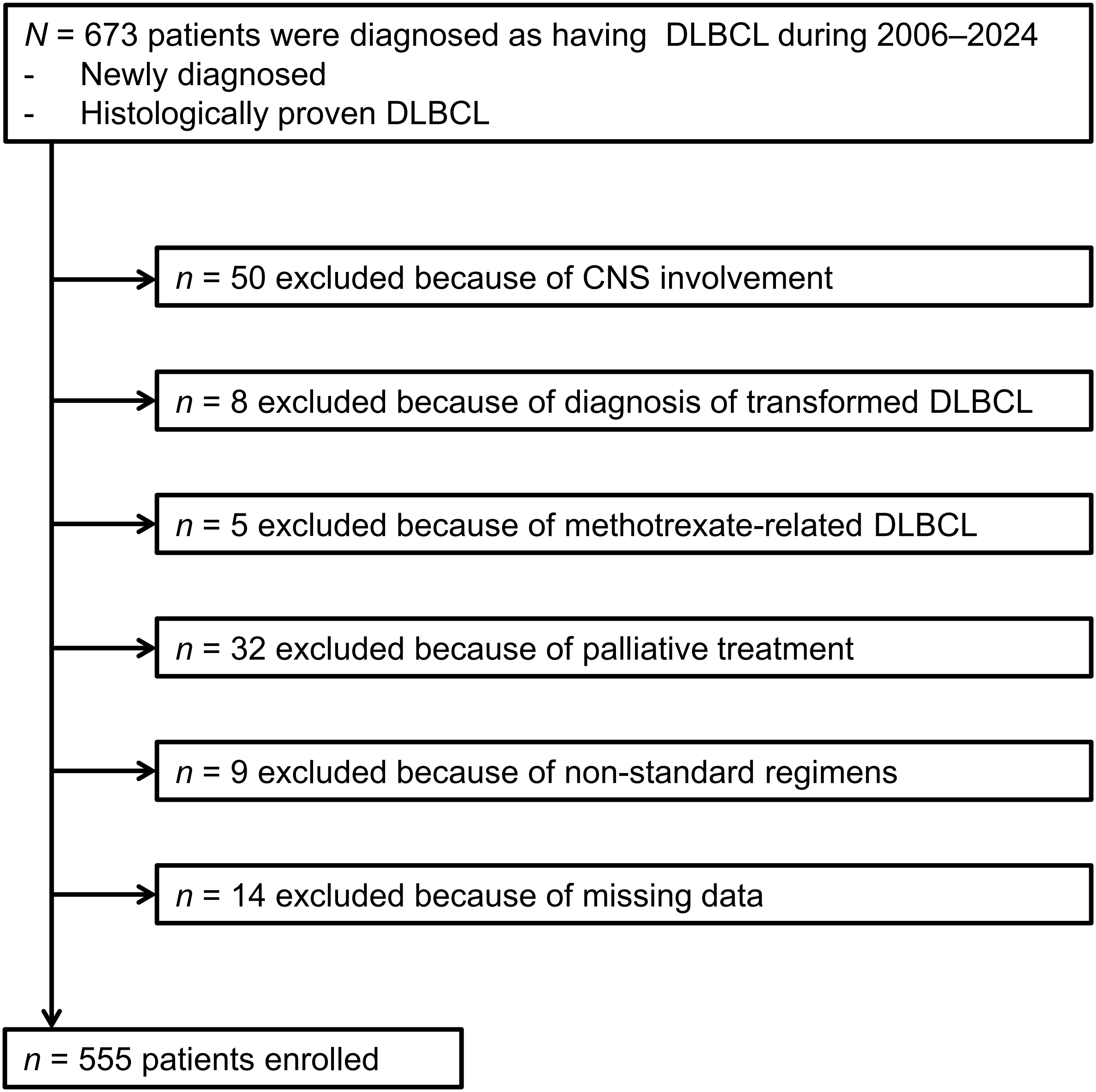

CNS = Central nervous system; DLBCL = Diffuse large B-cell lymphoma.

Supplementary figure 2. Receiver operating characteristic curve showing the performance of the GNRI for predicting the incidence of severe adverse events.

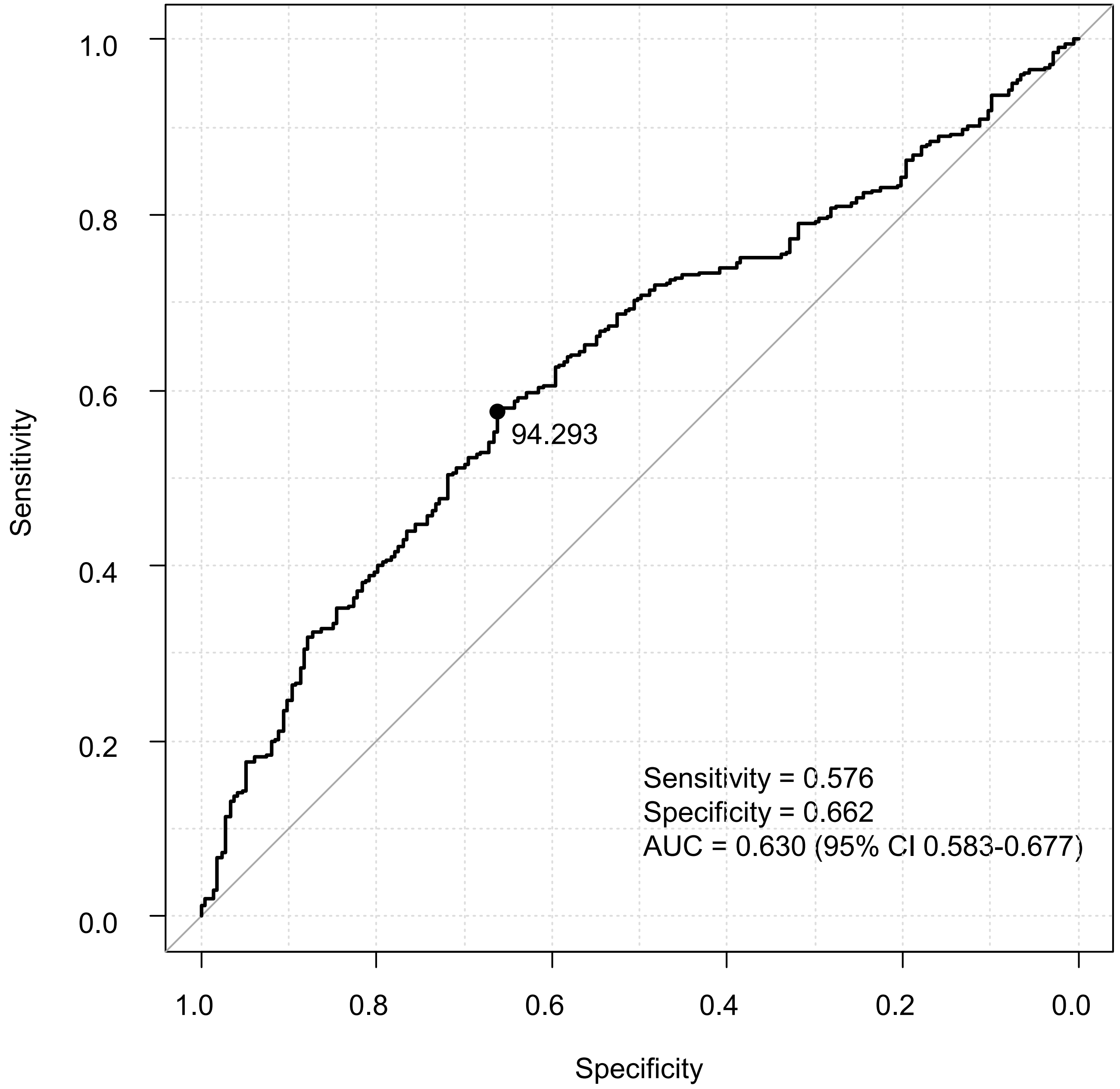

AUC = Area under the curve; CI = Confidence interval.

Supplementary figure 3. Covariable-adjusted logistic model with restricted cubic spline with showing the association between GNRI and the risk of severe adverse events according to age.

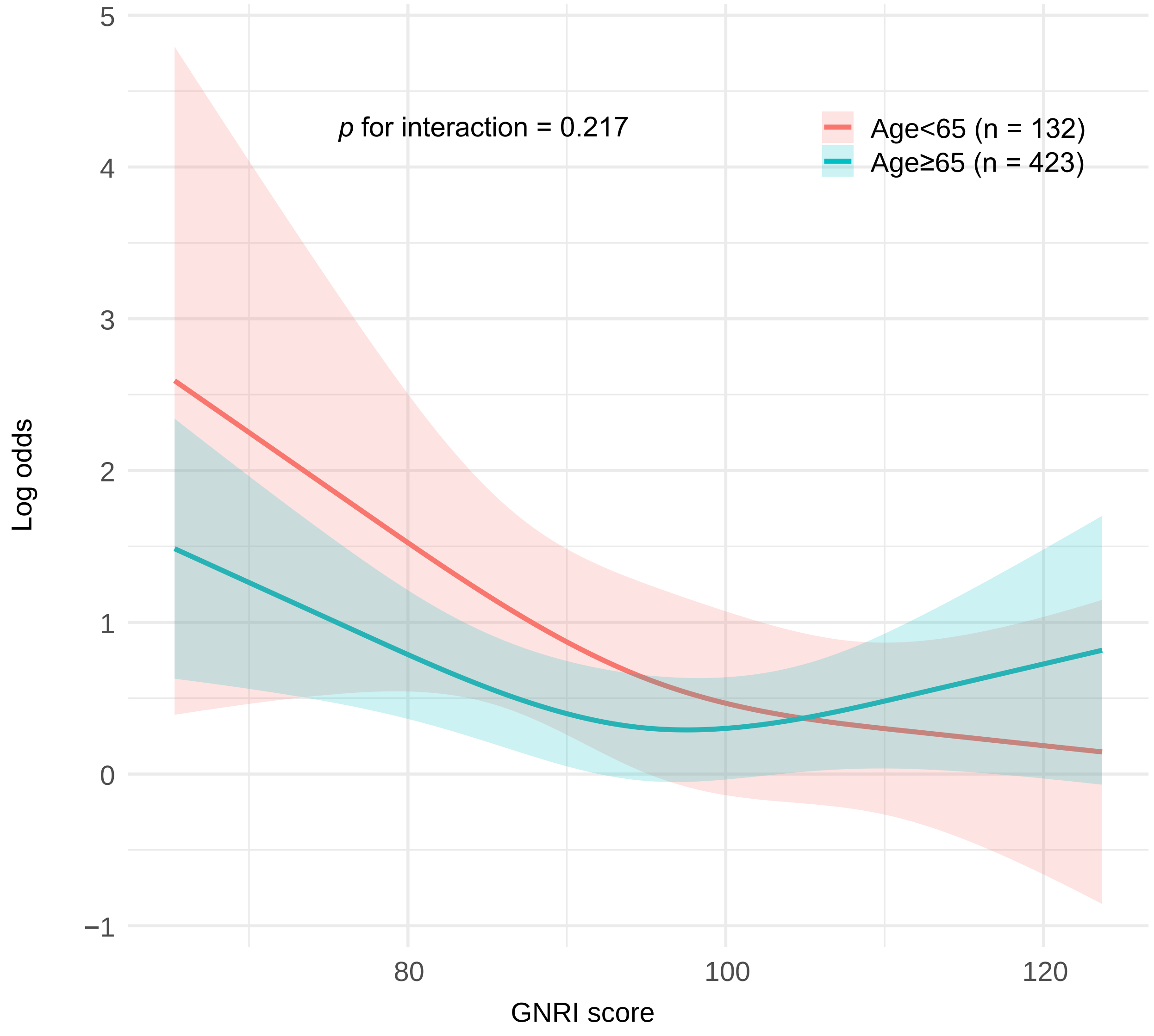

Solid line represents the log hazard ratio, shaded area is the 95% confidence interval. GNRI = Geriatric Nutritional Risk Index.

Supplementary figure 4. Forest plot showing odds ratios for severe adverse events associated with GNRI, stratified by age and initial treatment regimen.

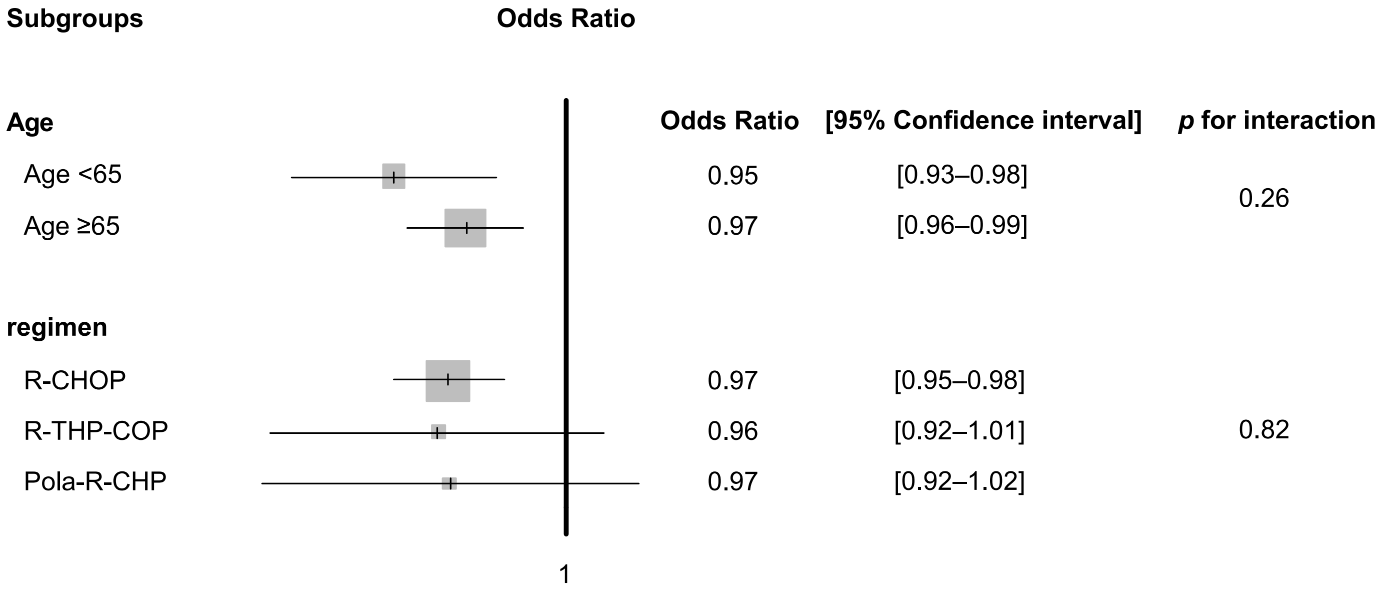

GNRI = Geriatric Nutritional Risk Index.

Supplementary figure 5. Calibration plot of the multivariable logistic regression model for predicting severe adverse events.

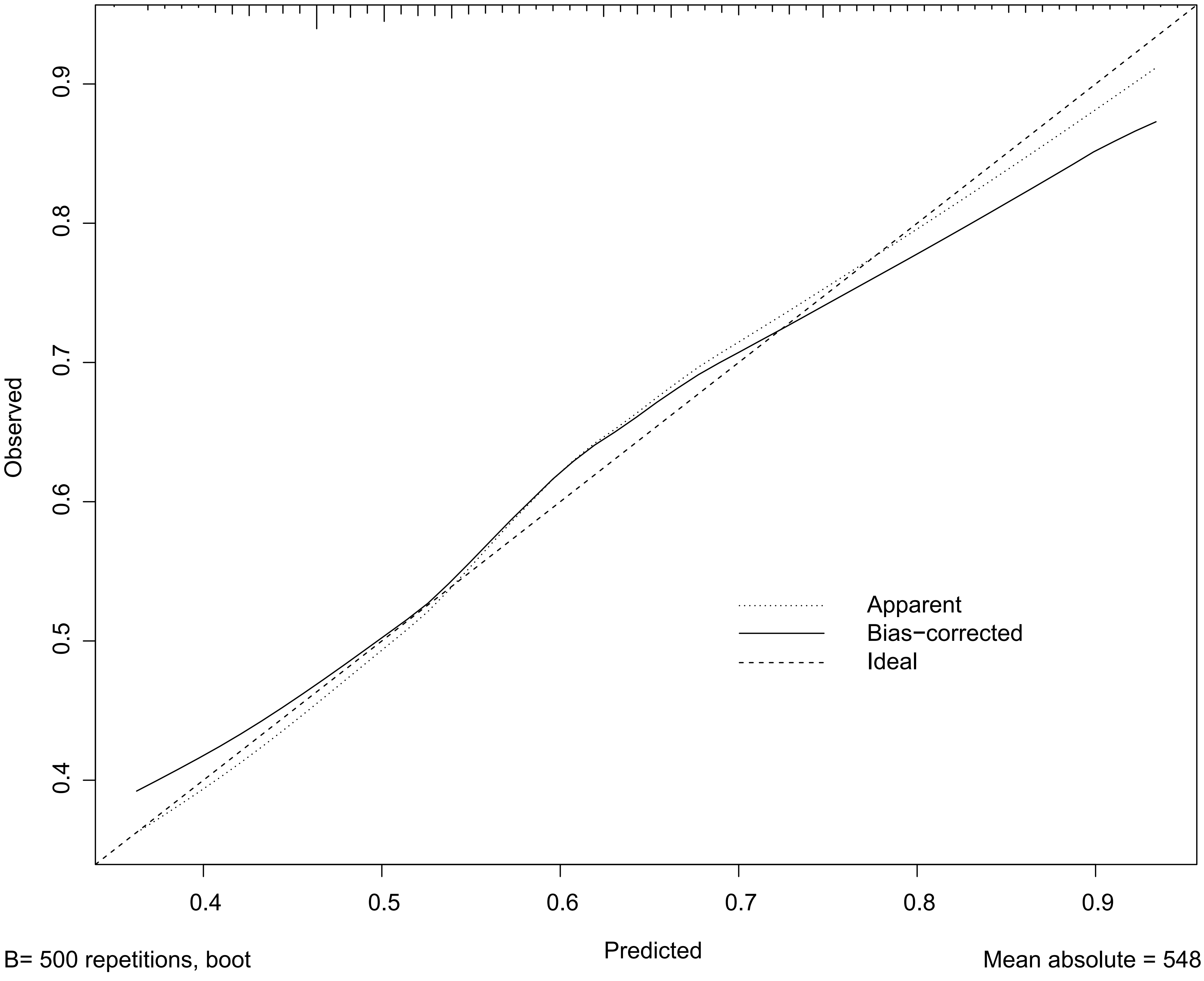

Supplementary figure 6. Decision curve analysis evaluating the incremental clinical utility of adding GNRI to the baseline prediction model for severe adverse events.

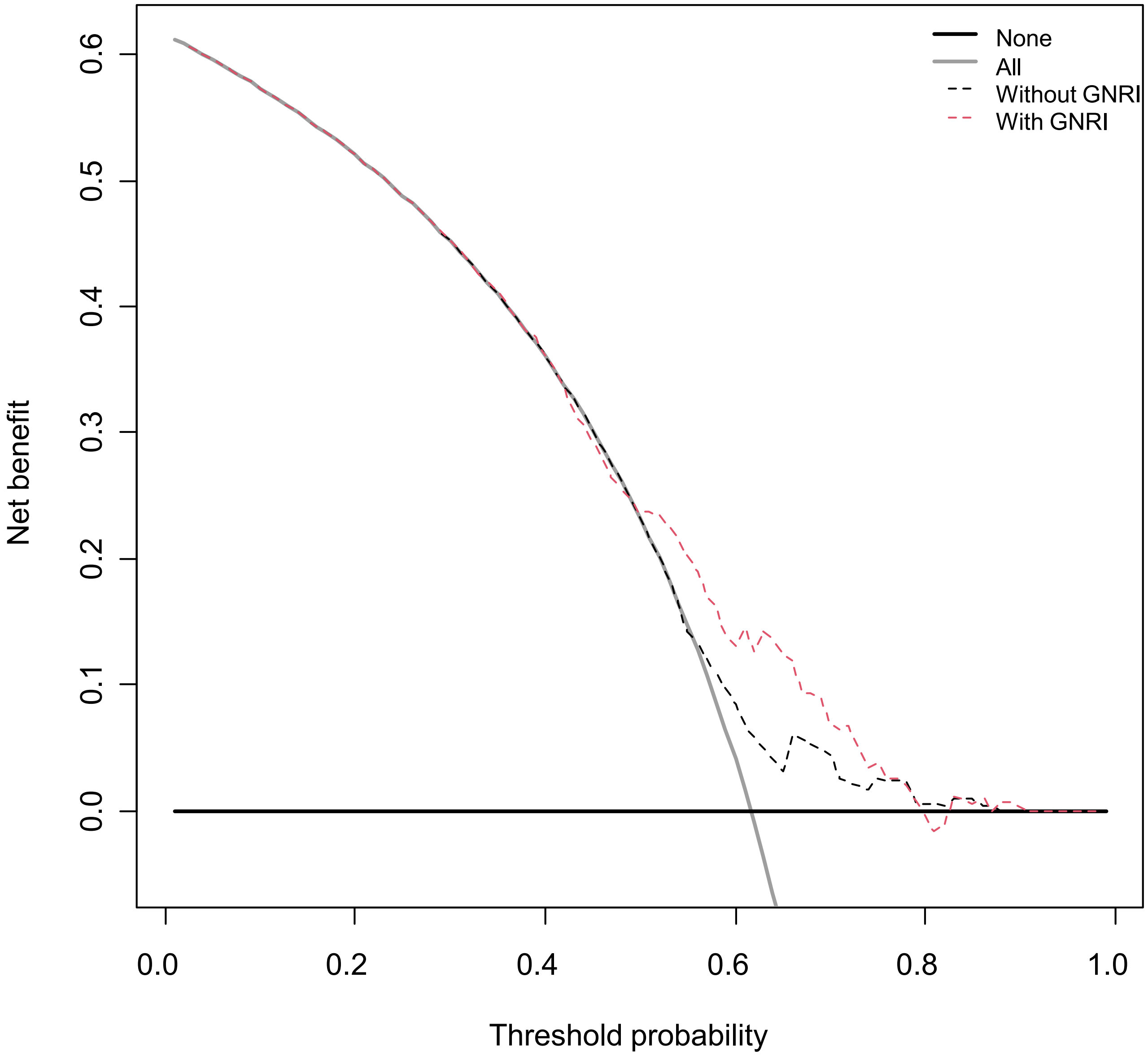

The analysis compares net benefit of models with and without GNRI across a range of threshold probabilities. The GNRI-inclusive model shows consistently higher net benefit, supporting its incremental clinical value.

GNRI = Geriatric Nutritional Risk Index.

Supplementary figure 7. Nomogram for predicting the probability of severe adverse events.

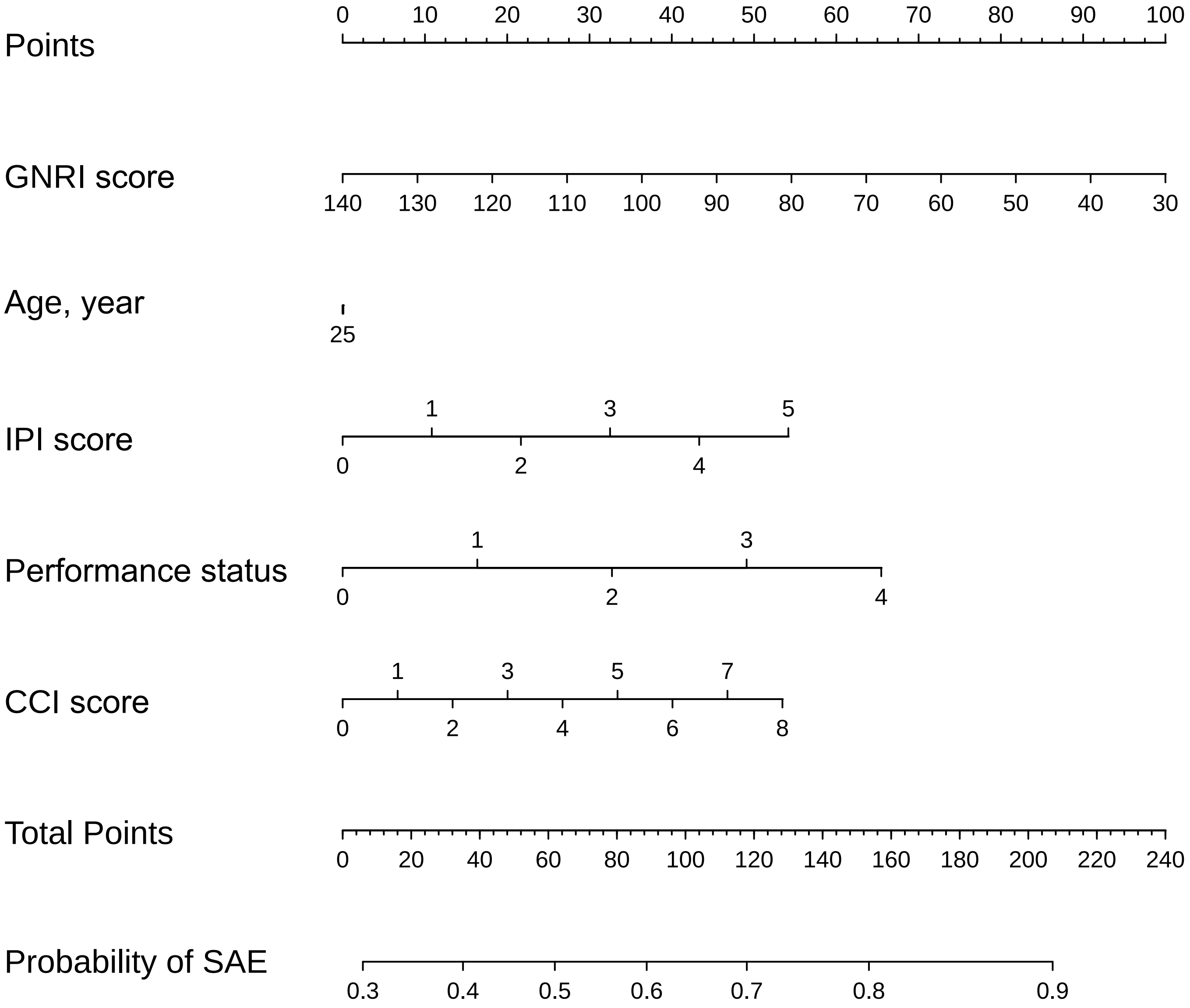

Total points derived from individual predictors correspond to the estimated risk of SAE during treatment. CCI = Charlson Comorbidity Index; GNRI = Geriatric Nutritional Risk Index; IPI = International Prognostic Index; SAE = Severe adverse event.

Supplementary table 1. Logistic regression analysis for clinical factors associated with the occurrence of severe adverse events.

	Univariable		Multivariable	
	OR (95% CI)	*p* value	OR (95% CI)	*p* value
Male	0.858 (0.609–1.210)	0.382	0.947 (0.655–1.370)	0.232
IPI, score	1.460 (1.290–1.660)	<0.001	1.300 (1.120–1.510)	<0.001
Bulky mass	1.530 (1.010–2.320)	0.047	1.100 (0.703–1.730)	0.671
CCI, score	1.250 (1.100–1.410)	<0.001	1.180 (1.030–1.340)	0.015
Total ARDI	0.997 (0.993–1.000)	0.305	1.000 (0.998–1.010)	0.274
GNRI score	0.967 (0.955–0.980)	<0.001	0.982 (0.967–0.997)	0.021

ARDI = average relative dose intensity; CCI = Charlson Comorbidity Index; CI = Confidence interval; GNRI = Geriatric Nutritional Risk Index; IPI = International Prognostic Index; OR = Odds ratio.

Supplementary table 2. Sensitivity analysis: Multivariable logistic regression model excluding total ARDI to assess potential post-treatment bias.

	Univariable		Multivariable	
	OR (95% CI)	*p* value	OR (95% CI)	*p* value
Male	0.858 (0.609–1.210)	0.382	0.944 (0.647–1.372)	0.760
IPI, score	1.460 (1.290–1.660)	<0.001	1.296 (1.116–1.505)	<0.001
Bulky mass	1.530 (1.010–2.320)	0.047	1.114 (0.711–1.745)	0.637
CCI, score	1.250 (1.100–1.410)	<0.001	1.160 (1.021–1.319)	0.023
GNRI score	0.967 (0.955–0.980)	<0.001	0.983 (0.968–0.998)	0.030

CCI = Charlson Comorbidity Index; CI = Confidence interval; GNRI = Geriatric Nutritional Risk Index; IPI = International Prognostic Index; OR = Odds ratio.
